# A novel mechanism of FTO modulating the progression of endometriosis through mediating the m6A methylation of GEF-H1 in a YTHDF1-dependent manner

**DOI:** 10.1186/s10020-025-01130-8

**Published:** 2025-02-25

**Authors:** Xin-Yu Ding, Hua-Ying Zhang, Jia-Hao Chen, Meng-Jie Yang, Zhi-Xiong Huang, Yi-Hong Lei, Qin-Kun Sun, Jian-Bin Bai, Dian-Chao Lin, Jian-Fa Lan, Lu-Lu Ren, Zheng-Yi Chen, Wei-Dong Zhou, Qiong-Hua Chen

**Affiliations:** 1 Laboratory of Research and Diagnosis of Gynecological Diseases of Xiamen City, Department of Obstetrics and Gynecology, the First Affiliated Hospital of Xiamen University, School of Medicine, Xiamen University, Xiamen, 361003 China; 2 National Institute for Data Science in Health and Medicine, Xiamen University, Xiamen, 361003 China; 3 The Third Clinical Medical College, Fujian Medical University, Fuzhou, 350000 China

**Keywords:** Endometriosis, Epigenetic modification, FTO, Invasion, Migration, m6A

## Abstract

**Background:**

Endometriosis (EMs) is a condition characterized by the growth of endometrial tissue outside the uterine cavity. Although this condition is benign, it has cancer-like features. N6-methyladenosine (m6A) is a common RNA modification involved in diverse biological processes, but its role in EMs remains unclear.

**Methods:**

A human endometrial stromal cell line (HESCs), primary eutopic endometrial stromal cells (Eu-ESCs), primary ectopic endometrial stromal cells (Ec-ESCs), and clinical samples were used in this study. A colorimetric assay was used to measure methylation levels in clinical and mouse EMs samples. Functional assays (CCK-8, EdU, Transwell, and wound healing) were used to evaluate phenotypic changes. m6A immunoprecipitation sequencing (MeRIP-seq) identified downstream targets. Mechanistic studies were conducted via qRT‒PCR, Western blot, RNA immunoprecipitation (RIP), dual-luciferase reporter, and RNA stability assays.

**Results:**

We detected aberrantly low levels of m6A within endometriotic lesions, which was attributed to increased expression of the m6A eraser fat mass and obesity-associated protein (FTO). Notably, estrogen and inflammatory factors, which are recognized as pathogenic agents in EMs amplify FTO expression while suppressing m6A levels. In vitro experiments demonstrated that overexpression of FTO in endometrial stromal cells leads to a reduction in m6A levels and concomitantly promotes their proliferation, migration, and invasion. Furthermore, both genetic deletion of *Fto* and chemical inhibition of FTO impeded the growth of ectopic endometrial lesions in vivo. By utilizing m6A-seq, we identified GEF-H1 (a Rho guanine nucleotide exchange factor) as a pivotal downstream target of FTO. Specifically, diminished m6A methylation at a certain site within the 3'UTR of GEF-H1 promotes its expression in a YTH N6-methyladenosine RNA-binding protein F1 (YTHDF1)-dependent manner, thereby activating the RhoA pathway. Subsequent experiments revealed that GEF-H1 mediates the effects of FTO in promoting migration and invasion.

**Conclusions:**

This study revealed that FTO decreases the m6A level of *GEF-H1*, thereby increasing its stability, which in turn activates the GEF-H1-RhoA pathway to promote the migration and invasion of endometrial stromal cells, thereby inducing EMs. Our findings suggest potential therapeutic avenues for targeting FTO to alleviate EMs progression.

**Supplementary Information:**

The online version contains supplementary material available at 10.1186/s10020-025-01130-8.

## Introduction

Endometriosis is a complex, chronic condition involving inflammation, hormonal dysregulation, immune responses, systemic effects, and diverse manifestations (Chapron et al. 2019; Taylor et al. 2021). This condition is defined as the shedding of active endometrial tissue outside of the uterine cavity (Zondervan et al. 2020; Allaire et al. 2023). Worldwide, approximately 190 million women suffer from endometriosis with typical symptoms, including severe pain, dysmenorrhea, dyspareunia and infertility, along with major impacts on their psychological health and quality of life (Simoens et al. 2012; Pluchino et al. 2016; Leeners et al. 2018; Horne and Missmer 2022). Cancer antigen-125 (CA-125) is a glycoprotein predominantly expressed in normal epithelial tissues of coelomic origin, such as those of the endometrium and peritoneum. Serum levels of CA-125 are frequently elevated in women diagnosed with Ems (Nisenblat et al. 2016). Serum anti-Müllerian hormone (AMH) levels are a reliable and valuable indicator of ovarian reserve and are often reduced in patients with EMs and inversely correlated with disease severity (Muzii et al. 2018). To date, regardless of the clinical approach used, approximately 50% of women treated for endometriosis have recurrent disease over a period of 5 years (Becker et al. 2017). Multiple mechanisms may cooperate to maintain the endometriosis phenotype, including endocrine disorders, epigenetic mechanisms, immunosuppression and metabolic alterations (Zhang et al. 2018; Marquardt et al. 2023; Samare-Najaf et al. 2024). However, the understanding of the molecular mechanisms involved in endometriosis development and therapeutic responses is still limited.

In recent years, considerable research efforts have been devoted to identifying RNA m6A modifications associated with various diseases. Similar to DNA modifications, regulatory alterations occur in RNA molecules, influencing their cellular functions (Niu et al. 2013; Liu and Pan 2016). Among these modifications, the N6-methyladenosine (m6A) modification is notable as the most prevalent post-transcriptional modification in RNA, representing a crucial epigenetic mechanism governing gene expression (Zhang et al. 2020; Huang et al. 2021). Approximately ten years ago, the discovery of the m6A mRNA demethylase fat mass and obesity-associated protein (FTO) revealed that m6A is a reversible modification (Jia et al. 2011). To date, researchers have identified three categories of m6A regulators: writers, erasers, and readers (Zaccara et al. 2019). Specifically, methyltransferase-like 3 and 14 (METTL3 and METTL14), along with other factors, constitute the m6A methyltransferase complex (MTC), which is responsible for installing m6A modifications on RNA as m6A writers (Wang et al. 2017), whereas FTO and alkB homolog 5 (ALKBH5) serve as erasers capable of removing m6A modifications (Yang et al. 2018). Consequently, the levels of m6A are dynamically regulated through the combined action of writers and erasers. The functional effects of m6A modifications are mediated by reader proteins that recognize this modification (Zhao et al. 2020). The YTHDF family, comprising YTHDF1, YTHDF2, and YTHDF3, plays a pivotal role in regulating the translation and stability of mRNAs, thereby influencing various biological processes (Patil et al. 2018; Chen et al. 2023). Additionally, members of the HNRNP family, including heterogeneous nuclear ribonucleoprotein A2/B1 (HNRNPA2B1), heterogeneous nuclear ribonucleoprotein C (HNRNPC), and heterogeneous nuclear ribonucleoprotein G (HNRNPG), can identify m6A modifications on mRNAs (Zhao et al. 2020). The binding of HNRNP proteins to m6A sites can be facilitated by structural alterations induced by m6A methylation.

Accumulating evidence suggests a causal link between variations in m6A levels and various diseases. For example, mutations in METTL14 and decreased expression of METTL3 in endometrial cancer have been shown to lower the m6A levels of critical AKT regulators (Liu et al. 2018). Consequently, this reduction fosters AKT pathway activation, leading to increased proliferation and tumorigenicity of endometrial cancer cells. Recent investigations have also shown that diminished m6A levels may contribute to the pathogenesis of endometriosis by increasing cellular migration and invasion (Li et al. 2021). Several studies have investigated the role of m6A modifications in EMs. For example, this modification has been shown to regulate the degradation of EZH2 and inhibit decidualization in endometriosis (Lin et al. 2024). Zhang reported that m6A alterations may be key factors in the progression of endometriosis, particularly the significant downregulation of METTL3 and YTHDF2 expression (Wang et al. 2023b). Another study reported that METTL3-mediated m6A methylation epigenetically regulates the progression of endometriosis through the METTL3-YTHDF2-SIRT1/FOXO3a axis (Wang et al. 2023a). However, the precise mechanisms by which m6A methylation influences cellular function, as well as the underlying pathways implicated in these alterations within the context of endometriosis, remain to be fully elucidated.

In this study, we aimed to elucidate the role of m6A RNA modification in the pathogenesis and progression of endometriosis and to explore the underlying molecular mechanisms involved. Initially, we confirmed that the upregulation of the m6A eraser FTO in endometriosis led to a decrease in m6A levels in ectopic endometrial tissues. Through mechanistic investigations employing both overexpression and knockdown techniques, we further demonstrated that FTO could increase the proliferation, migration, and invasion of human endometrial stromal cells (HESCs) in vitro, whereas the inhibition of FTO reduced the growth of ectopic lesions in vivo. Additionally, m6A-seq analyses revealed that GEF-H1, the Rho guanine nucleotide exchange factor, is a crucial target mRNA of FTO involved in mediating the migration and invasion of HESCs. On the basis of the findings presented herein, we identify critical roles for FTO-mediated m6A modifications in the progression of endometriosis.

## Materials and methods

### Patients and cell culture

Seventeen samples of normal endometria were collected under hysteroscopy from patients without endometriosis who visited for tubal infertility, and endometritis and intrauterine polyps were excluded. Sixty-one patients suffering from ovarian endometriotic cysts provided 61 ectopic endometria and 29 eutopic endometria. All the clinical samples were obtained from the Department of Gynecology and Obstetrics, the First Affiliated Hospital of Xiamen University. The use of clinical specimens received permission from the hospital's ethics committee, and all patients provided informed consent.

Human endometrial stromal cells (HESCs) (CRL 4003™) were obtained from ATCC and cultured in DMEM/F12 (1:1) supplemented with 10% carbon-adsorbed fetal bovine serum (FBS), 1% penicillin–streptomycin, 1% insulin, transferrin, selenium, ethanolamine solution (ITS-X), and 500 ng/mL puromycin at 37 °C with 5% CO_2_. All the HESCs were confirmed as not containing mycoplasma by short tandem repeat profiling.

Primary cells were isolated from the eutopic endometria and endometriotic lesions of patients with endometriosis (EMs). Briefly, endometrial tissues were minced into small pieces approximately 1 mm in size. These tissue fragments were then incubated in collagenase IV solution for 1 h and DNase I solution for 30 min to dissociate single endometrial cells. Following digestion, the tissue suspension was sequentially filtered through 150 μm and 24 μm nylon cell strainers to separate epithelial and stromal cells. The isolated cells were then cultured in DMEM/F12 medium supplemented with 10% FBS, 1% penicillin–streptomycin and 1% ITS in a 5% CO_2_ incubator at 37 °C.

### Reagents

DMEM/F12 (1:1) (#l340KJ; BasalMedia, China), carbon-adsorbed fetal bovine serum (#CMS003.02; CellMax, China), ITS (51500056; Thermo Fisher Scientific, USA), puromycin (#S250J0, BasalMedia, China) and penicillin–streptomycin (#15140; Gibco, USA) were used to culture the cells. IL-1β (#HY-P78459), Estradiol (#HY-B0141), LPS (#HY-D1056) and Rhein (#HY-N0105) were obtained from MCE (USA). The anti-GAPDH mouse monoclonal antibody (#40493) was procured from ABclonal (Boston, USA). FTO (#ab126605), METTL14 (#ab220031), GEF-H1 (#ab155785), RhoA (#ab187027), YTHDF1 (#ab252346), Rac1 (#ab155938), and Cdc42 (#ab41429) antibodies were obtained from Abcam (Cambridge, UK).

### Database source and processing

The expression profiles of m6A regulators were obtained from publicly available datasets, including GEO (https://www.ncbi.nlm.nih.gov/geo/) and ArrayExpress (https://www.ebi.ac.uk/arrayexpress/). Four datasets—GSE7305, GSE7307, GSE51981, and E-MTAB-694—were selected for analysis. All datasets were generated using the GPL570 platform ([HG-U133_Plus_2] Affymetrix Human Genome U133 Plus 2.0 Array). The raw data (CEL files) were imported and normalized using the robust multiarray average (RMA) algorithm implemented in the “affy” package in R. The datasets included 10 EU and 10 EC samples from GSE7305; 23 EU and 17 EC samples from GSE7307; 34 NM and 77 EU samples from GSE51981; and 17 EU and 18 EC samples from E-MTAB-694. These datasets were integrated into R to create a merged matrix containing 34 NM, 127 EU, and 45 EC samples from the GPL570 platform. Batch effects were adjusted using the “removeBatchEffect” function in the “limma” package in R.

### Hormone and inflammatory factor treatment

HESCs were cultured in phenol red-free DMEM/F12 without FBS for 24 h to remove endogenous steroids. The cells were treated with different concentrations of E2 (#HY-B0141, MCE, USA), IL-1β (HY-P78459, MCE, USA) and LPS (HY-D1056, MCE, USA) for 48 h to analyze the expression of m6A regulators and the levels of m6A modification. The cells were transferred to medium containing 0.01% DMSO and used as vehicle controls.

### Genetically modified cell line establishment

Lentiviruses carrying shNC, oeNC (negative control), shFTO, oeFTO for humans were purchased from GeneChem. The sequences of the shRNAs used were as follows: *FTO*, 5′-TCACCAAGGAGACTGCTATTT-3′. The target cells were infected with lentivirus at an MOI of 10 for 18 h.

The *GEF-H1* overexpression and mutation were ordered from Genechem. siRNAs used in this research were obtained from RiboBio. Sequences for siRNAs are the following: *YTHDF1*, 5′-CCGCGTCTAGTTGTTCATGAA-3′; *GEF-H1*, 5′-UUUAAGAGAUCGUAGGCAA-3′. Transfection was achieved by using Lipofectamine RNAiMAX (Invitrogen) for siRNA, or Lipofectamine 3000 (Invitrogen) for the plasmids following manufacturer’s protocols.

### Quantitative real—time polymerase chain reaction (qRT-PCR)

Total RNA was isolated using RNAiso Plus (#9108; TaKaRa Biotechnology; Kyoto, Japan) according to the manufacturer’s instructions and analyzed by NanoDrop. cDNA was synthesized via using a PrimeScript RT reagent kit (#9108; TaKaRa Biotechnology; Kyoto, Japan). qRT‒PCR was performed using TB Green Premix Ex Taq II (#RR820A, TaKaRa) in a LightCycler 480 system (Roche Molecular Biochemicals, Mannheim, Germany). The results were normalized to the human GAPDH mRNA level. The sequences of primers used in qRT‒PCR are as follows: *METTL14*_ For, AGTGCCGACAGCATTGGTG; *METTL14*_ Rev, GGAGCAGAGGTATCATA-GGAAGC; *METTL3*_For, CTATCTCCTGGCACTCGCAAGA; *METTL3*_Rev, GCTTGAACCGTGCAACCACATC; *YTHDF1*_For, CAAGCACACAACCTCCA-TCTTCG; *YTHDF1*_Rev, GTAAGAAACTGGTTCGCCCTCAT; *YTHDF2*_For, TAGCCAGCTACAAGCACACCAC; *YTHDF2*_Rev, CAACCGTTGCTGCAG-TCTGTGT; *FTO*_For, CCAGAACCTGAGGAGAGAATGG; *FTO*_Rev, CGATG-TCTGTGAGGTCAAACGG; *ALKBH5*_For, CCAGCTATGCTTCAGATCGCCT; *ALKBH5*_Rev, GGTTCTCTTCCTTGTCCATCTCC; *GEF-H1*_For, TCCCTCA-TTGACGAAGCAGA; *GEF-H1*_Rev, GGTGCAGCTCTGTCTGGATT.

### Western blot

The protein samples were separated by 10% SDS‒PAGE and transferred onto PVDF membranes (#03010040001; Roche, Basel, Switzerland). Different primary antibody dilutions and corresponding HRP-labeled secondary antibodies were incubated with the membranes. The dilution ratios of the antibodies used in the experiments were as follows: FTO (1:10,000), METTL14 (1:1000), GEF-H1 (1:1000), RhoA (1:500), Rac1 (1:1000), Cdc42 (1:10,000), YTHDF1 (1:1000), GAPDH (1:10,000), and secondary antibodies (1:10,000). The signal was ultimately visualized via enhanced chemiluminescence (ECL).

### m6A RNA methylation quantification (colorimetric)

The m6A RNA methylation of total RNA was quantified via an m6A RNA methylation quantification kit (ab185912; Abcam). Two hundred nanograms of RNA was coated on the wells containing the assay mixture with binding buffer and incubated for 90 min at 37 °C. Then, diluted capture antibody (1:1000) solution was added to the wells after washing, and the samples were incubated for 60 min at room temperature. Diluted detection antibody solution (1:2000) and diluted enhancer solution (1:5000) were then added to the assay wells separately and incubated for 60 min at room temperature. Washing the wells was required between each step. Finally, after 100 μL of developer solution was added to each well in the dark for 10 min, stop solution was added to stop the reaction. The m6A levels were quantified colorimetrically by reading the absorbance of each well at a wavelength of 450 nm, and then, calculations were performed on the basis of the standard curve.

### m6A immunoprecipitation

Total RNA (400 μg) was extracted from the cells. The RNA was purified via TRIzol, and the RNA extraction protocol was followed. The RNA was fragmented via fragmentation reagents (AM8740; Thermo Fisher Scientific) following the manufacturer’s protocols. One hundred microliters of mRNA fragmented with RNasin, DTT (dithiothreitol) (1 M), m6A antibody and 5 × immunoprecipitation buffer (IPP) were rotated overnight at 4 °C. The RIP mixture was mixed with 40 μL of Protein G beads and rotated for 3 h at 4 °C. The beads were washed three times with NaCl, Nonidet P-40 (NP-40), or adenosine-uridine-cytosine-guanosine (AUCG). Diethyl pyrocarbonate (DEPC) water, DTT (1 M), m6A antibody and 5 × IPP were added to the beads, which were vibrated for 1 h at 37 °C, and then, the supernatant was removed. Two hundred microliters of RNA with NaAc (sodium acetate), glycogen blue and ethanol were stored at − 80 °C overnight. The RNA was centrifuged at 15,000 × *g* and 4 °C for 20 min and then washed with 75% ethanol two times. DEPC water was added to the RNA, which was subsequently analyzed by a NanoDrop system.

### MeRIP-seq

The m6A-modified mRNAs were sequenced using MeRIP-seq at Novogene (Beijing, China). A total of 200 µg of RNA was extracted from the cells, and its integrity and concentration were assessed using an Agilent 2100 Bioanalyzer and a SimpliNano spectrophotometer. Fragmented mRNA (~ 100 nt) was incubated with an anti-m6A polyclonal antibody (Synaptic Systems, catalog no. 202003) at 4 °C for 2 h during immunoprecipitation. The immunoprecipitated mRNA or input RNA was used for library construction with the NEBNext® Ultra™ II RNA Library Prep Kit (New England Biolabs, E7770). Sequencing was performed on an Illumina platform (paired-end, 150 bp), with 3 independent biological replicates.

The raw sequencing data in FASTQ format were processed using fastp (v0.19.11) to remove adapters, poly-N sequences, and low-quality reads, yielding high-quality clean data. Quality metrics, including the Q20, Q30, and GC content, were calculated. Clean reads were aligned to the reference genome using BWA (v0.7.12) after the genome index was constructed. Peaks were identified using the exomePeak R package (v2.16.0), with input samples used as controls and a q value threshold of 0.05. m6A-enriched motifs were analyzed using HOMER (v4.9.1).

The peak annotation linked the identified peaks to genes, with a focus on exonic regions. Differential peak analysis was performed using the exomePeak R package, with a P value < 0.05 and a fold change > 1. Genes associated with differential peaks were subjected to Gene Ontology (GO) and Kyoto Encyclopedia of Genes and Genomes (KEGG) enrichment analyses.

### RNA immunoprecipitation (RIP)

RIP was conducted via an EZ-Magna RIP RNA-Binding Protein Immunoprecipitation Kit (17-70, Sigma, US). The cell pellet was an equal pellet volume of complete RIP lysis buffer. The mixture was incubated on ice for 5 min. YTHDF1 and IgG antibodies were incubated with protein A/G magnetic beads for 30 min at room temperature. The cell lysate was incubated with the washed magnetic beads at 4 °C overnight in RIP immunoprecipitation buffer. Then, RNA was extracted with phenol:chloroform:isoamyl alcohol 25:24:1 reagent (516726, Sigma, US), and the expression of the target genes was determined via RT‒qPCR.

### Luciferase reporter assays

After transfection for 48 h, the cells were collected and washed twice with PBS. The culture medium was removed by aspiration, and an appropriate volume of 1 × cell lysis buffer was added to the cells. The lysates were incubated at room temperature for 5 min with gentle shaking, followed by thorough pipetting. The lysates were then centrifuged at 12,000 × *g* for 20 min at room temperature, and the supernatants were collected for subsequent luciferase activity measurements. Luciferase activities were measured using the Dual Luciferase Reporter Assay Kit (DL101-1; Vazyme Biotech, China) according to the manufacturer’s instructions. For the firefly luciferase assay, 100 μL of equilibrated Luciferase Substrate was added to reaction tubes or microplate wells, followed by the addition of 20 μl of cell lysate supernatant. The mixture was mixed quickly and immediately measured using a luminometer or microplate reader. Subsequently, 100 μL of freshly prepared Renilla substrate working solution was added to the same reaction mixture, mixed quickly, and immediately measured to determine Renilla luciferase activity.

### mRNA stability assay

Each sample was harvested at 0, 3, and 6 h after treatment with 5 mg/mL actinomycin D (HY-17559, MCE, US). Total RNA was isolated with an RNAiso Plus. The RNA concentrations were determined via RT‒qPCR.

### Migration and invasion assays

After being transfected for 48 h and then cultured in serum‐free DMEM/F12 for 12 h, 1 × 10^5^ cells suspended in DMEM/F12 containing 0.1% bovine serum albumin (BSA) were plated into 8 μm pore size upper chambers (#3422; Corning; US). For Invasion assays, the upper chambers were coated with Matrigel (10 μg/insert, BD Biosciences no. 356234) in DMEM/F12 (1:1) and dried for 6 h. For the Migration assays, the upper chambers were not coated. The lower chamber was filled with 700 μl of complete DMEM/F12 containing 10% BSA and maintained in an incubator at 37 °C for 48 h. The upper chambers were subsequently fixed with 4% paraformaldehyde and stained with crystal violet solution, followed by three washes with PBS. The migrated cells on the lower surface of the upper chambers were counted and captured with an EVOS M7000 Cell Imaging System (AMF7000, Thermo Fisher Scientific, US) at 20 × magnification and were detected in 5 independent fields.

### Cell proliferation assay

A total of 1 × 10^4^ HESCs with modified genes were seeded into 96-well plates in DMEM/F12 medium. CCK-8 reagent (#HY-K0301; MedChemExpress, China) was added to each well, and the mixture was allowed to react for 2 h at 37 °C. The absorbance of the supernatants was measured with an ELISA reader spectrophotometer (Dynatec Laboratories, VA).

### EdU labeling assays

EdU labeling assays were performed as described in the instructions of the EdU assay kit (#C0071S; Beyotime Biotechnology, China). A total of 1 × 10^3^ genetically modified cells were plated in 96-well plates. After treatment with the EdU solution, the cells were fixed and permeabilized, and the Click-iT additive solution (prepared according to the instructions) was added to incubate the cells for 30 min in the dark. Finally, Hoechst 33,342 was used to stain the nucleus. The results were observed and captured using an EVOS M7000 Cell Imaging System (AMF7000, Thermo Fisher Scientific, USA).

### Immunohistochemistry (IHC)

The slides were routinely dewaxed, dehydrated, subjected to antigen retrieval, and then incubated with primary antibodies (FTO, 1:200, GEF-H1, 1:500) at 4 °C overnight. The slides were washed and incubated with the corresponding secondary antibodies (1:100). Finally, a DAB color development kit (#AR1022; Boster Biological Technology, Wuhan, China) was used to stain the slides. The results were observed and captured with an EVOS M7000 Cell Imaging System (AMF7000, Thermo Fisher Scientific, USA).

### Endometriotic mouse model assay

Female BALB/c and C57BL/6 J mice (6–8 weeks old, 15–20 g) were purchased from the Animal Research Laboratory of Xiamen University and housed under specific pathogen-free conditions. Female *Fto*-floxed C57BL/6 J mice (*Fto*^f/f^; NM-CKO-190005) were obtained from Model Organisms (Shanghai, China). All animal experiments were approved by the Institutional Animal Care and Use Committee and conducted in compliance with ethical guidelines for animal research. For elimination of endogenous estrogen (E2), all the mice were ovariectomized before the experiments.

For establishment of a uterine-specific FTO knockout model, *Fto*-floxed C57BL/6 J mice were divided into two groups. The experimental group (n = 3) was administered Cre-adenovirus (Cre-AVV; Genechem, China) via intrauterine injection to induce uterine-specific deletion of FTO, whereas the control group (n = 3) was injected with a control adenovirus (control-AVV). Each mouse received a single intrauterine injection of 4 × 10^11^ viral genomes (20 μl). Two weeks post-injection, uterine tissues were collected to confirm the knockout efficacy via quantitative PCR and Western blot analysis. Both Cre-AVV-treated (*Fto*-knockout) and control-AVV-treated *Fto*-floxed mice were subsequently used as donor mice to generate EMs models. Uterine tissues were harvested, fragmented into small pieces (~ 2 mm^3^), and transplanted into the abdominal cavities of C57BL/6 J recipient mice to establish the EMs model.

For the drug intervention model, BALB/c mice were used. The mice were divided into three groups: donor mice (n = 5), normal saline-treated endometriosis model mice (EMs + NS; n = 5), and Rhein-treated endometriosis model mice (EMs + Rhein; n = 5). Uterine tissues from donor mice were harvested, fragmented, and transplanted into the abdominal cavities of BALB/c recipient mice to establish the EMs model. The EMs + Rhein group received intraperitoneal injections of 1 mL of normal saline containing Rhein (10 mg/mL) every three days, whereas the EMs + NS group received 1 mL of normal saline alone.

All the mice were monitored for one month to assess the growth of the ectopic endometrial lesions. At the end of the experiment, the ectopic lesions were harvested, and their size and weight were measured for further analysis.

### Statistical analysis

All the data were analyzed with GraphPad Prism 9 software (San Diego, CA, USA). Comparisons of the data were performed via Student’s t test or one-way analysis of variance (ANOVA). A P value < 0.05 was considered to indicate statistical significance.

## Results

### Abnormal m6A modification is observed, and FTO is upregulated in EMs

**Fig. 1:**
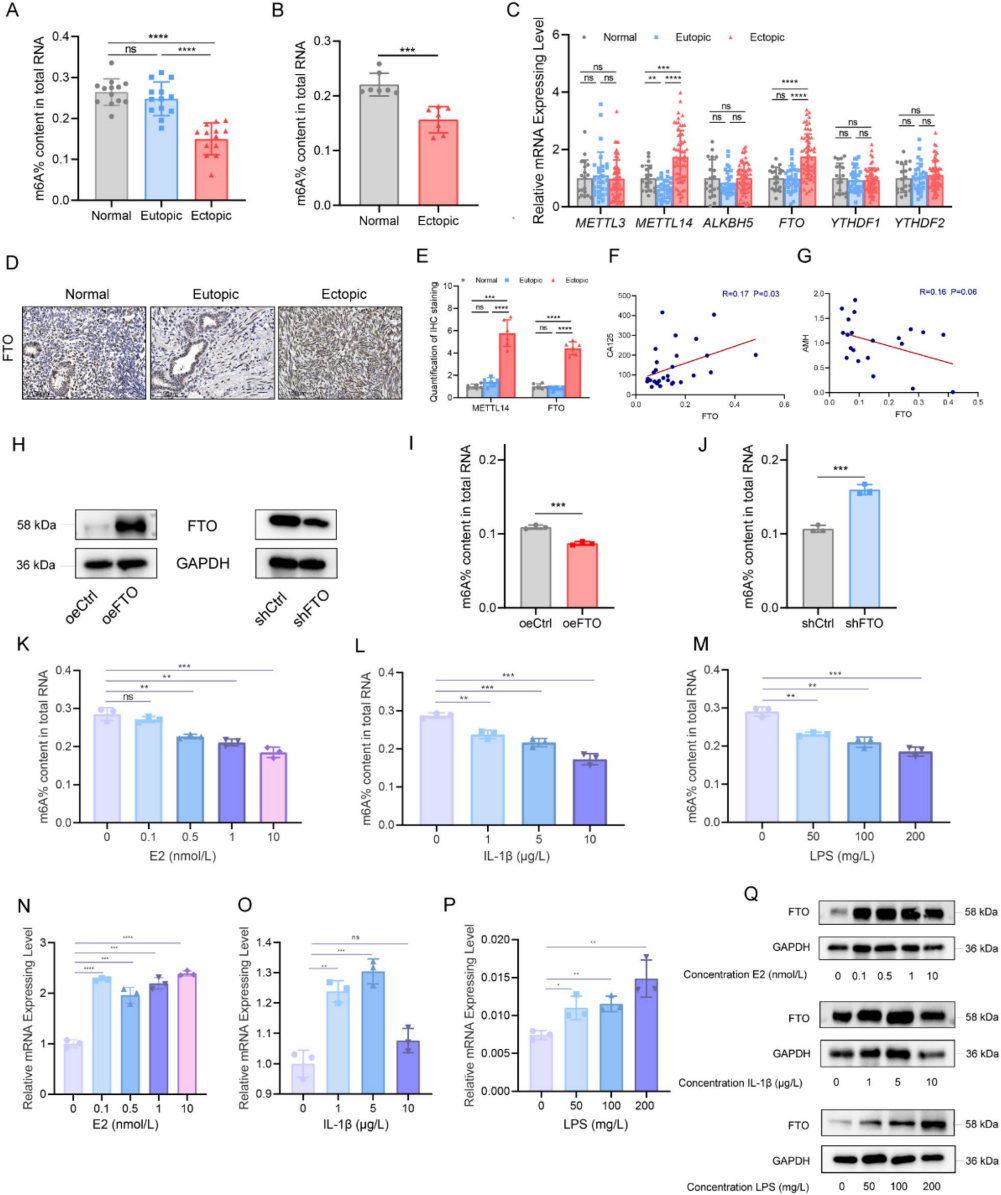
Abnormal m6A modification is observed, and FTO is upregulated in Ems. **A** The m6A content of total RNA in normal endometrial tissues (Normal; n = 13), eutopic endometrial tissues (Eutopic; n = 13) and paired ectopic endometrial tissues (Ectopic; n = 13) from endometriosis patients. ****P < 0.0001. **B** The m6A content of total RNA in normal endometria from control mice and ectopic endometria from EMs model mice (n = 7). ***P < 0.001. **C** Examination of m6A modification-related gene expression in Normal (n = 17), Eutopic (n = 29) and Ectopic (n = 61) samples via qPCR. *P < 0.05, **P < 0.005, ***P < 0.001, ****P < 0.0001. **D**, **E** IHC staining of the FTO protein in Normal (n = 6), Eutopic (n = 6), and Ectopic (n = 6) tissues (**D**), with corresponding quantification of the IHC results (**E**). Scale bars: 150 μm. ***P < 0.001, ****P < 0.0001. **F** Scatter plot showing the correlation of the expression of *FTO* in the ectopic group with the serum CA125 level (n = 29). R = 0.17, P = 0.03. **G** Scatter plot illustrating the correlation between FTO expression in ectopic lesions and serum AMH levels (n = 21). R = 0.16, P = 0.06. **H** Overexpression and knockdown efficacy of *FTO* at the protein level in HESCs. **I**, **J** m6A levels of total RNA in HESCs in which FTO was overexpressed or knocked down. ***P < 0.001. **K**‒**M** m6A levels of total RNA in HESCs treated with estrogen, IL-1β or LPS. **P < 0.005, ***P < 0.001. **N**‒**P** qPCR analysis of *FTO* expression in HESCs treated with various concentrations of estrogen (**N**), IL-1β (**O**) or LPS (**P**). *P < 0.05, **P < 0.005, ***P < 0.001, ****P < 0.0001. **Q** Immunoblotting assay of FTO expression in HESCs treated with estrogen, IL-1β or LPS

To investigate whether alterations in m6A modifications are implicated in endometriosis, we initially compared the total RNA m6A levels among normal endometrial tissues (Normal), paired eutopic endometrial tissues (Eutopic), and ectopic endometrial tissues (Ectopic) using a colorimetric m6A quantification strategy. Strikingly, we observed a decrease in m6A levels in the ectopic tissues compared with their paired eutopic counterparts and normal tissues (Fig. 1A). However, no significant difference in m6A levels was noted between the eutopic and normal tissues. These findings establish a global reduction in m6A levels in RNA across endometriosis tissues.

We subsequently established a mouse model of endometriosis to further investigate the role of m6A modifications in vivo. Ectopic lesions were successfully implanted into recipient BALB/c mice (Figure S1A-B), and after 30 days, we collected ectopic lesions (n = 7) and control endometria (n = 7) to analyze the RNA-m6A levels. Consistent with the trend observed in the clinical endometriosis samples, we found that m6A levels were lower in the ectopic lesions than in the control endometria (Fig. 1B), indicating that the mouse model of endometriosis exhibited changes similar to those observed in human disease.

As m6A modification is reversible and regulated by writers and erasers, we conducted a systematic analysis of the transcriptomic levels of m6A modulators in relevant samples obtained from public databases, which included 34 normal, 127 eutopic, and 45 ectopic samples (GSE7305, GSE7307, GSE51981, and E-MTAB-694). This analysis revealed differential expression of m6A-related factors in the normal, eutopic, and ectopic samples (Figure S1C). Importantly, the data revealed that the demethyltransferase *FTO* was upregulated in the ectopic samples compared with the eutopic and normal samples. To independently validate this finding, we collected endometrial tissues from healthy individuals (n = 17), as well as eutopic (n = 29) and ectopic endometria from patients with endometriosis (n = 61), to evaluate the expression of m6A regulators, including *METTL3*, *METTL14*, *ALKBH5*, *FTO*, *YTHDF1*, *and YTHDF2*, via qRT‒PCR (Fig. 1C). Our results demonstrated that the methyltransferase *METTL14*, along with *FTO*, was significantly upregulated in ectopic tissue. Conversely, there were no significant differences in the expression levels of *METTL3*, *ALKBH5*, *YTHDF1*, or *YTHDF2*. Furthermore, through immunohistochemistry, we confirmed that FTO was increased in ectopic tissues (Fig. 1D, E), indicating that the observed increase in mRNA levels corresponded to elevated protein levels. Given that serum CA125 levels are commonly used to reflect the severity of EMs and that serum AMH levels indicate the impact of EMs on ovarian function, we analyzed the correlation between FTO expression levels in the ectopic lesions of EMs patients and serum CA-125 and AMH levels. The analysis revealed a weak positive correlation between FTO expression levels in the ectopic lesions of EMs patients and serum CA-125 levels (R = 0.17, P = 0.03; Fig. 1F). A weak negative correlation was observed with the serum AMH level, but this association was not statistically significant (R = 0.16, P = 0.06; Fig. 1G). These weak correlations suggest that while FTO expression may be associated with serum marker levels, other factors likely contribute to these relationships. We individually manipulated the expression of FTO in HESCs by overexpressing it and inhibiting its expression using lentivirus (Fig. 1H). We found that the total m6A levels in HESCs increased upon FTO knockdown but decreased upon FTO overexpression (Fig. 1I, J).

Previous studies have shown that EMs is an estrogen (E2)-dependent and inflammation-related condition (Horne and Missmer 2022). E2 and inflammatory factors promote the proliferation, migration, invasion, and angiogenesis of endometrial cells while also inhibiting apoptosis and inducing inflammation (Wu et al. 2010; Han et al. 2015; Peng et al. 2020). Thus, we investigated whether E2 and inflammatory factors affected the expression of FTO and the m6A level in HESCs. We treated HESCs with varying concentrations of E2 (0–10 nmol/L), interleukin-1 beta (IL-1β; 0–10 μmol/L), and lipopolysaccharide (LPS; 1–200 mg/L) for 48 h. Our results revealed that E2 treatment led to a reduction in m6A levels in HESCs (Fig. 1K). Similarly, treatment with IL-1β and LPS also resulted in decreased m6A levels in HESCs (Fig. 1L-M). Furthermore, qRT‒PCR analysis demonstrated that FTO expression increased following treatment with E2, IL-1β, and LPS (Fig. 1N‒P). Additionally, we observed the upregulation of FTO at the protein level in the HESCs treated with E2, IL-1β, and LPS (Fig. 1Q).

Taken together, our findings suggest that FTO is the primary factor driving aberrant m6A modifications in endometriosis and is involved in its pathological processes.

### FTO regulation of m6A is associated with the proliferation, migration, and invasion of HESCs

**Fig. 2:**
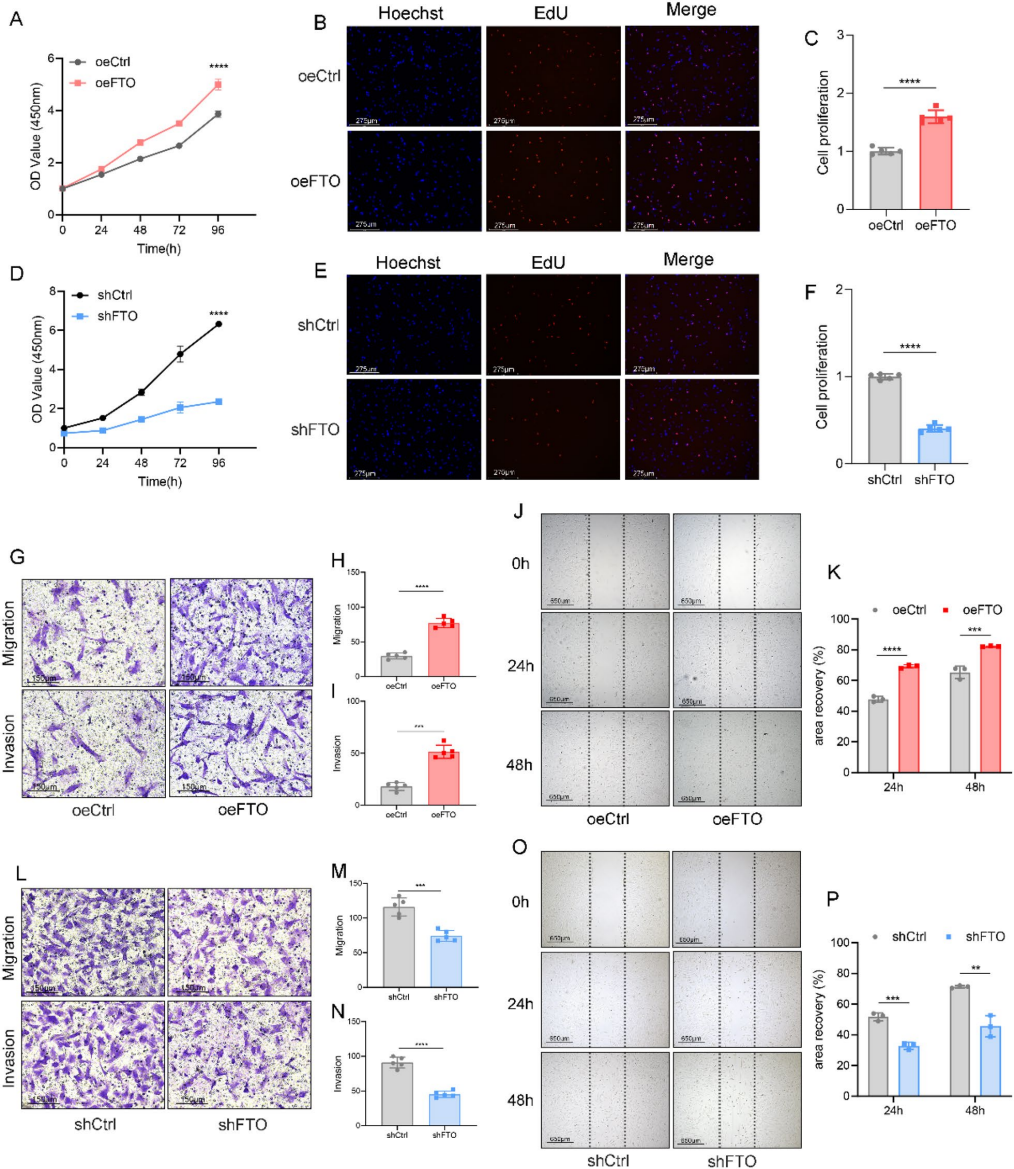
FTO regulation of m6A is associated with the proliferation, migration, and invasion of HESCs. **A**–**C** Cell proliferation was measured by CCK-8 (**A**) and EdU (**B**) assays in oeCtrl (negative control) and oeFTO (overexpressing FTO) HESCs. Scale bars: 275 μm. **C** Quantification of the results of the EdU assay. ****P < 0.0001. **D**–**F** Cell proliferation was measured by CCK-8 (**D**) and EdU (**E**) assays in shCtrl (negative control) and shFTO (knockdown of FTO) HESCs. Scale bars: 275 μm. **E** Quantification of the results of the EdU assay. ****P < 0.0001. **G**–**I** Transwell migration and invasion assays of FTO-overexpressing and control HESCs. The results of the quantification of cell migration and invasion are presented in (**H**, **I)**. ***P < 0.001, ****P < 0.0001. Scale bars: 150 μm. **J**, **K** A wound healing assay was performed on HESCs overexpressing *FTO*. A scratch was created, and the width was measured 24 h and 48 h after the scratch. ***P < 0.001, ****P < 0.0001. Scale bars: 650 μm. **L**‒**N** The invasive and migratory abilities of HESCs transfected with lentiviruses carrying shFTO were explored via a transwell assay. Cells exhibiting migration/invasion are presented in the histogram in (**M**, **N**). ***P < 0.001, ****P < 0.0001. Scale bars: 150 μm. **O**, **P** Representative images and quantification of the migration of FTO-knockdown HESCs or their corresponding controls. **P < 0.005, ****P < 0.0001. Scale bars: 650 μm

Next, we investigated how the decreased m6A RNA levels induced by FTO affect the biological functions associated with EMs in HESCs. To this end, we stably overexpressed and silenced FTO in HESCs and observed its effects on proliferation, migration, and invasion. The overexpression of FTO increased proliferation, whereas the knockdown of FTO suppressed proliferation in HESCs (Fig. 2A–F). Similarly, the migration and invasion of HESCs were promoted by FTO overexpression and inhibited by FTO knockdown (Fig. 2G–P).

To confirm that our findings extend beyond the HESC line, we assessed the effects of FTO knockdown and overexpression in eutopic endometrial stromal cells (Eu-ESCs) and ectopic endometrial stromal cells (Ec-ESCs). We observed similar m6A-mediated changes in cell proliferation, migration, and invasion (Figure S2), confirming the consistency of our results across different cell types associated with endometriosis.

In summary, these findings collectively suggest that the reduction in m6A mRNA methylation induced by FTO in endometriosis promotes the proliferation, migration, and invasion of endometrial stromal cells. These findings underscore the critical role of m6A in the progression of endometriosis and highlight its potential as a therapeutic target for this condition.

### Knockout of *Fto* inhibits ectopic lesion growth in EMs model mice

**Fig. 3:**
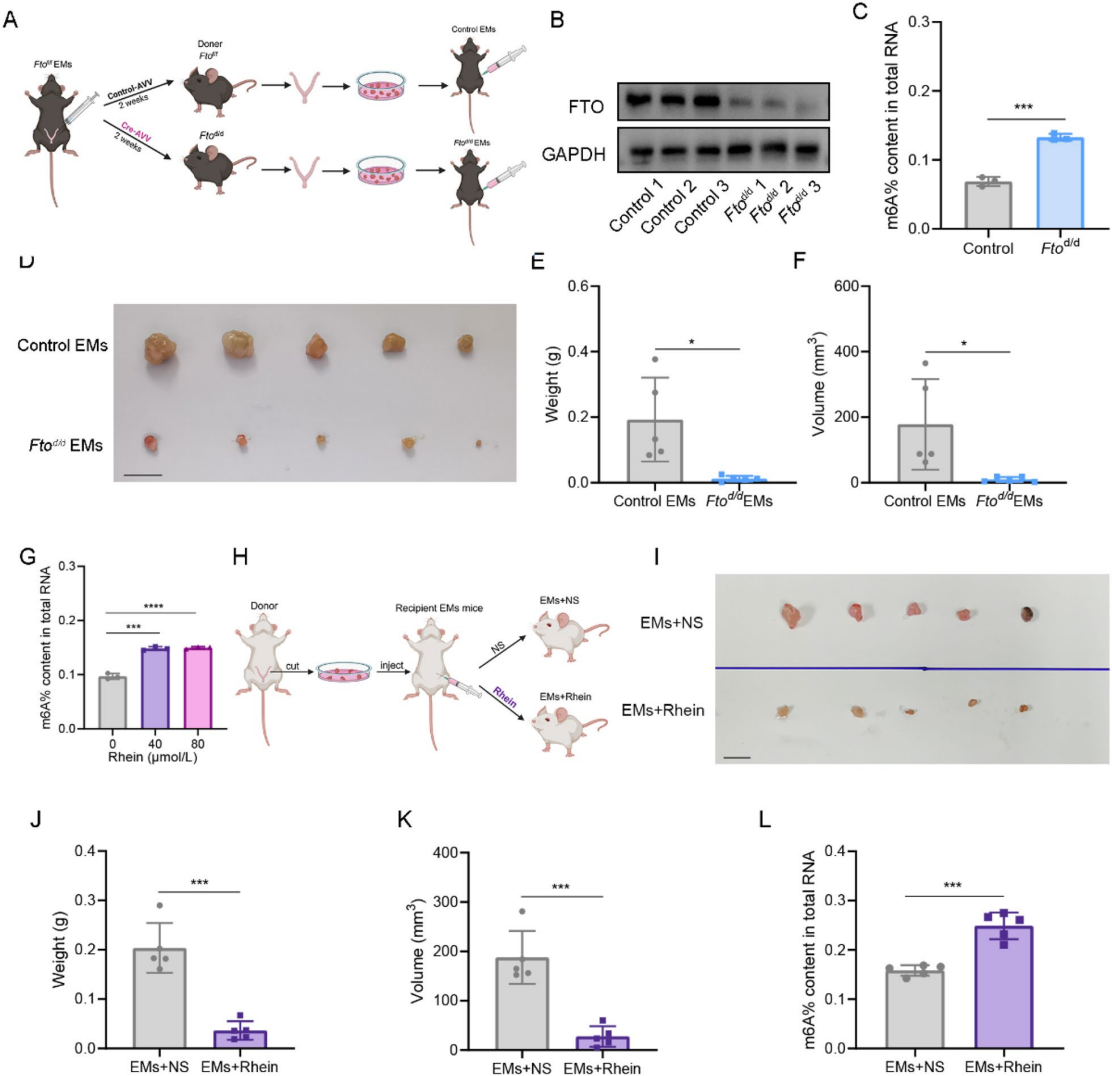
Knockout of *Fto* inhibits ectopic lesion growth in EMs model mice. **A** Schematic diagram of the EMs mouse model constructed by uterine-specific knockout of* Fto*. **B** The expression of FTO in the uteri of control and *Fto*^d/d^ mice (n = 3). **C** m6A levels in the uteri of control and *Fto*^d/d^ mice. ***P < 0.001. **D** Representative image of ectopic lesions excised from control EMs and *Fto*^d/d^ EMs mouse models (n = 5). **E**, **F** Ectopic lesion weights (**E**) and ectopic lesion volumes (**F**) determined after excision from mice in the abovementioned groups. *P < 0.05. (G) m6A levels in HESCs in which FTO was inhibited by Rhein at various concentrations. ***P < 0.001, ****P < 0.0001. **H** Schematic diagram of the Rhein intervention experiment in the mice with EMs. **I** Inhibition of FTO effectively suppressed ectopic lesion growth in mice (n = 5). EMs + NS: NS-injected endometriosis group; EMs + Rhein: Rhein-injected endometriosis group. **J**, **K** Weights (**J**) and volumes (**K**) of ectopic lesions in the EMs and EMs + Rhein groups. ***P < 0.001. **L** m6A levels of ectopic lesions in the EMs and EMs + Rhein groups. ***P < 0.001

To determine the potential causal role of FTO-mediated m6A regulation in the development of endometriosis in vivo, we generated mice with uterine deletion of *Fto* (*Fto*^d/d^) by injecting *Fto* floxed C57BL/6 J mice (*Fto*^f/f^) with Cre-AVV through the uterus (Fig. 3A). We then measured the protein levels of FTO and m6A levels in the uteri of the *Fto*^d/d^ mice. The results revealed a decrease in FTO expression levels and an increase in m6A levels in the uteri of the *Fto*^d/d^ mice (Fig. 3B, C). *Fto*^d/d^ mice were used as donor mice to generate EMs model mice, and one month later, the size and weight of the ectopic tissue were measured. Figure 3D–F demonstrates that knocking out *Fto* leads to significant inhibition of ectopic lesion growth in mice, as evidenced by significantly lower weights and volumes of ectopic lesions in the *Fto*^d/d^ EMs model mice than in the control mice.

Rhein, a competitive binding inhibitor of FTO, was used in this assay (Chen et al. 2012). We first treated HESCs with Rhein and determined the m6A total RNA levels to confirm the inhibitory effect of Rhein. As expected, Rhein treatment led to an increase in the total m6A level in HESCs (Fig. 3G). The mice whose endometrial tissues were implanted were subsequently intraperitoneally injected with Rhein at a dose of 10 mg/kg every three days for one month, whereas the control group received normal saline (NS) treatment (Fig. 3H). Our results demonstrated that Rhein treatment effectively suppressed the growth of ectopic lesions in vivo, as shown by significant reductions in the weights and volumes of the ectopic lesions compared with those in the EMs+NS group (Fig. 3I–K). Furthermore, we compared the m6A levels in ectopic lesions between the Rhein treatment group and the NS control group. As depicted in Fig. 3L, the ectopic lesions of the Rhein treatment group presented higher m6A levels than those of the EMs+NS group. Overall, these findings indicate that FTO inhibits the formation of ectopic lesions through the regulation of m6A modification levels.

### MeRIP-seq identifies GEF-H1 as a downstream target of FTO-mediated m6A modification

**Fig. 4:**
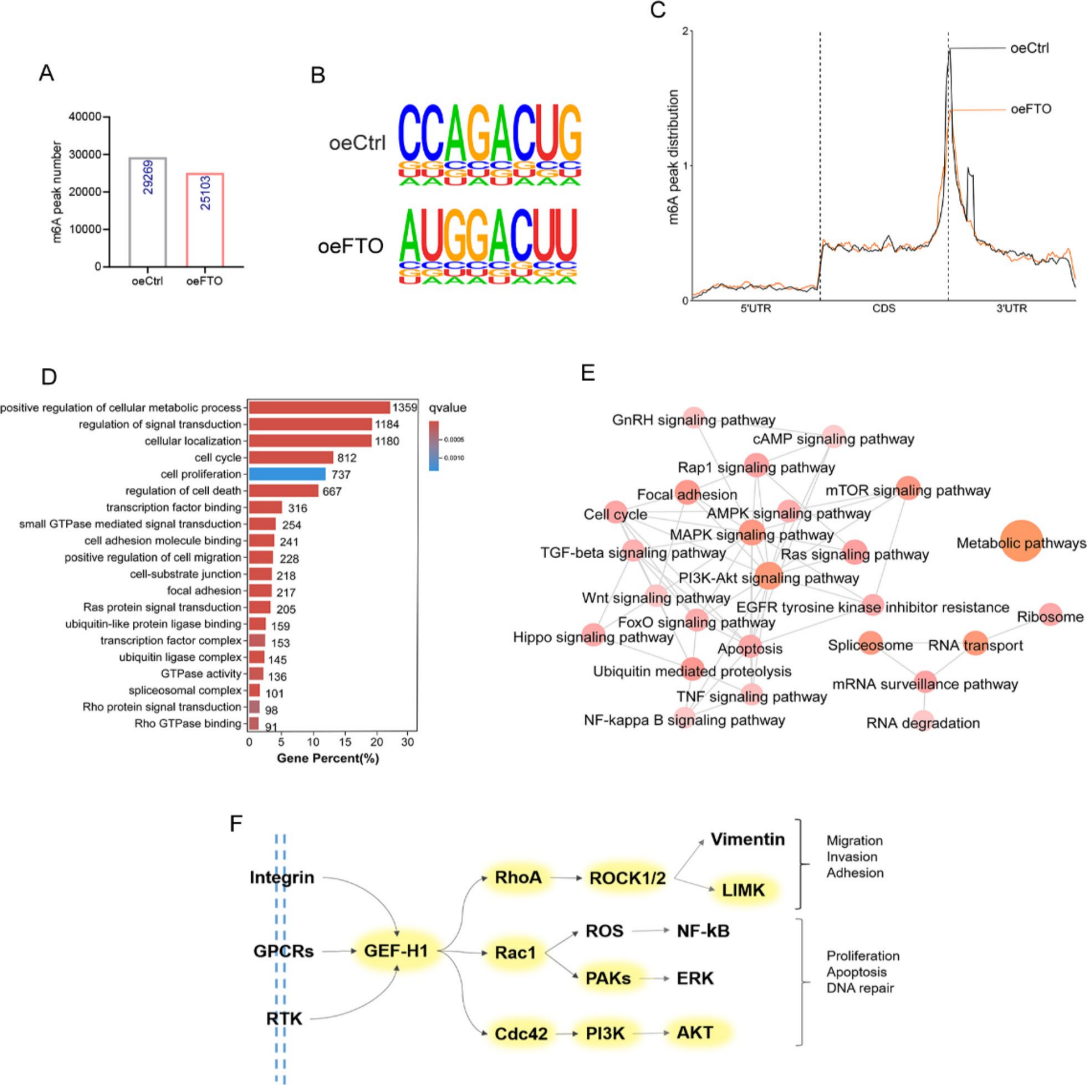
MeRIP-Seq identifies GEF-H1 as a downstream target of FTO-mediated m6A modification. **A** The number of m6A peaks detected in ctrl and oeFTO HESCs. **B** m6A motif identified in HESCs with or without FTO overexpression. **C** Distribution of m6A peaks across the length of mRNAs. **D**, **E** GO enrichment analysis (**D**) and KEGG analysis (**E**) of transcripts with reduced m6A in ctrl versus oeFTO HESCs. (**F**) Diagram of the Rho pathway with genes affected by m6A marked in yellow. The diagram is based on KEGG annotations

To clarify the underlying molecular mechanisms of the involvement of FTO in EMs, particularly to identify its target genes, we next performed MeRIP-seq. Three pairs of stable FTO-overexpressing and control HESCs were sequenced, yielding 29,269 and 25,103 m6A peaks in the control and FTO-overexpressing HESCs, respectively (Fig. 4A). Like previous m6A modification studies, we observed that m6A peaks were enriched for the typical DRACH motif (D = A/G/U, R = A/G, H = A/C/U) (Fig. 4B). Moreover, the enrichment of m6A peaks near the 3'UTR in the FTO-overexpressing HESCs was lower than that in the control cells (Fig. 4C).

We subsequently conducted Gene Ontology (GO) and Kyoto Encyclopedia of Genes and Genomes (KEGG) analyses on the MeRIP-seq data. GO analysis revealed that m6A differential transcripts were associated with crucial biological processes, such as signal transduction, cell metabolism, RNA splicing, and transcriptional regulation, all of which are essential for the development of endometriosis (Fig. 4D). Notably, both the GO and KEGG analyses highlighted the enrichment of pathways related to GTPases and the Rho family (Fig. 4D, E). Our previous investigations indicated that Ras homolog family member A (RhoA) is upregulated in endometriosis and is intricately linked to the E2/ERα/ERK signaling pathway, thereby promoting epithelial‒mesenchymal transition (EMT) and proliferation, ultimately contributing to endometriosis development (Huang et al. 2020).

Hence, we propose that decreased m6A methylation might facilitate endometriosis progression through the Rho family pathway. Indeed, our analysis revealed significant alterations in m6A modifications within genes associated with the Rho family pathway according to KEGG pathway annotation subsequent to FTO overexpression. These genes included *GEF-H1*, an upstream activator of RhoA, as well as downstream effectors such as *ROCK1/2*, *RAC1*, *Cdc42*, *PI3K*, *AKT*, and *LIMK* (Fig. 4F). Collectively, these findings suggest that GEF-H1 serves as a downstream target of FTO.

### FTO increases GEF-H1 expression in an m6A-YTHDF1-dependent manner

**Fig. 5:**
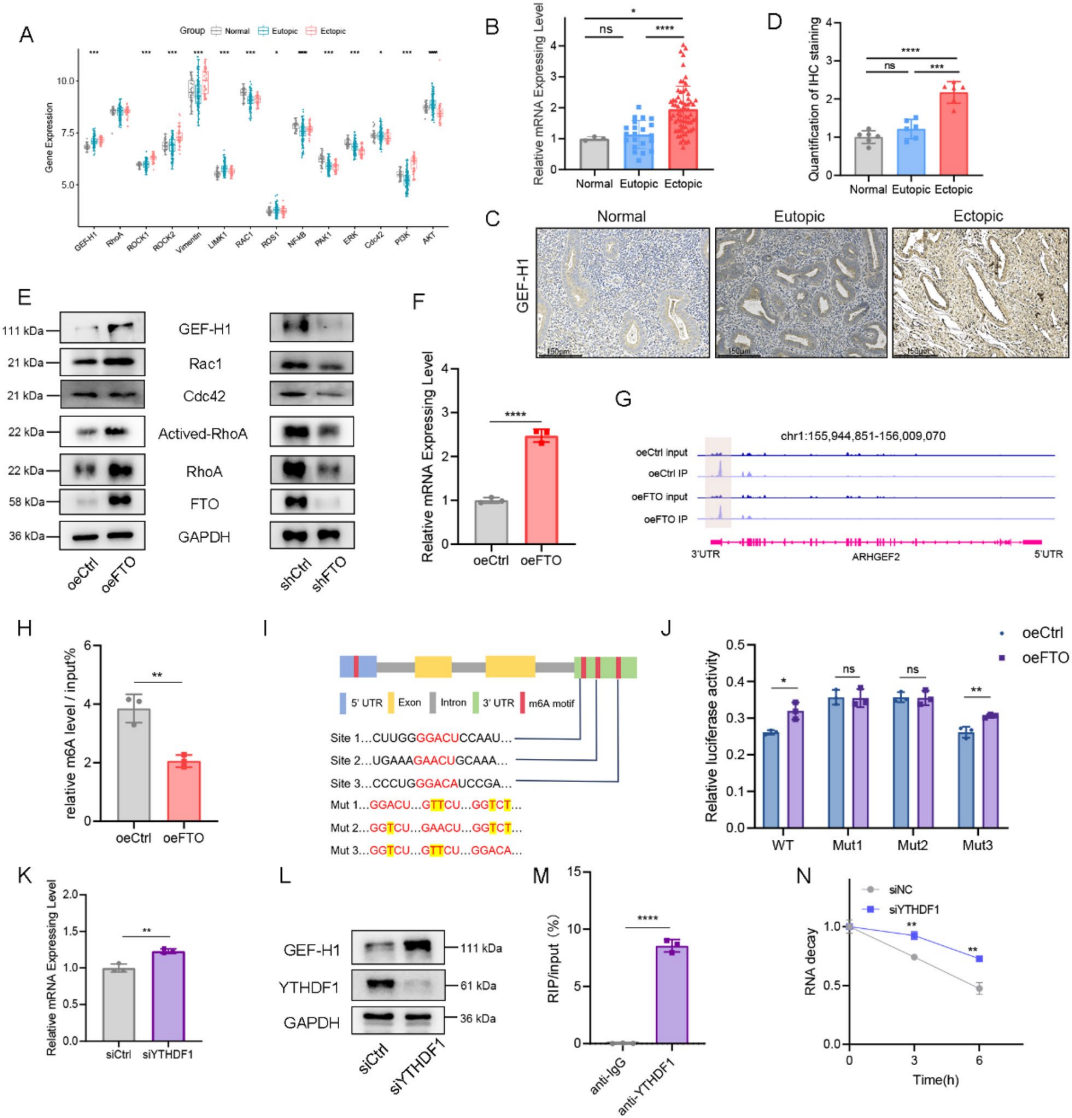
FTO-mediated m6A regulates GEF-H1 through YTHDF1. **A** Box plot of Rho family gene expression in Normal (n = 34), Eutopic (n = 127) and Ectopic (n = 45) samples. *P < 0.05, **P < 0.005, ***P < 0.001, ****P < 0.0001. **B** *GEF-H1* expression levels in Normal (n = 3), Eutopic (n = 21), and Ectopic (n = 67) tissues were analyzed by qPCR. *P < 0.05, ****P < 0.0001. **C**, **D** IHC staining (**C**) and quantitative analysis (**D**) of GEF-H1 expression in Normal (n = 6), Eutopic (n = 6), and Ectopic (n = 6) tissues. Scale bars: 150 μm. ***P < 0.001, ****P < 0.0001. **E** Immunoblot analysis of the expression of GEF-H1 and downstream genes in oeFTO and shFTO HESCs. **F** RT‒qPCR was used to quantify the relative mRNA levels of *GEF-H1* transcripts in oeCtrl and oeFTO HESCs. ****P < 0.0001. **G** The average read density from MeRIP-seq on oeCtrl HESCs and oeFTO HESCs shows the m6A peaks identified in the *GEF-H1* transcripts. **H** MeRIP was used to quantify the relative m6A level of *GEF-H1*. ***P < 0.001. **I** Mutations in m6A consensus sequences were generated by replacing adenosine with thymine. **J** Relative luciferase activity of the wild-type and 3-mutant *GEF-H1* 3'UTR reporter vectors in FTO-overexpressing HESCs. *P < 0.05, **P < 0.005, ***P < 0.001. **K** *GEF-H1* expression was significantly upregulated at the RNA level in YTHDF1-knockdown HESCs. **P < 0.005. **L** Immunoblot analysis of the levels of GEH-H1 in HESCs upon transient siRNA knockdown of YTHDF1. ***P < 0.001, ****P < 0.0001. **M** RIP-qPCR was used to assess the association of the *GEF-H1* transcript with the YTHDF1 protein. ****P < 0.0001. **N** RNA lifetime for *GEF-H1* in HESCs transfected with control siRNA or siRNA targeting YTHDF1 was determined by monitoring transcript abundance after transcriptional inhibition. **P < 0.005

We delved deeper into the relationship between FTO-mediated m6A modifications and their regulatory effects on the expression or translation of GEF-H1 in endometriosis. Initially, we scrutinized the expression of Rho family genes within pertinent array data (GSE7305, GSE7307, GSE51981, and E-MTAB-694). Notably, our analysis revealed increased expression levels of GEF-H1 mRNA in the ectopic group (Fig. 5A). This trend was observed in *GEF-H1* mRNA levels through qRT‒PCR analysis (Fig. 5B) and was further confirmed at the protein level via immunohistochemical staining of clinical EMs tissues (Fig. 5C‒D).

We subsequently employed genetic manipulations to investigate the impact of FTO levels on GEF-H1. Our findings indicated that FTO overexpression augmented GEF-H1 expression, whereas FTO knockdown hindered GEF-H1 expression in HESCs (Fig. 5E). Furthermore, the activity and expression of RhoA and RAC1 were increased in FTO-overexpressing HESCs, whereas these measures were decreased following FTO knockdown (Fig. 5E). Moreover, overexpression of FTO promoted the mRNA expression of *GEF-H1* (Fig. 5F). MeRIP-seq revealed that transcripts encoding *GEF-H1* presented decreased m6A methylation in the 3'UTR of oeFTO HESCs (Fig. 5G). To validate that GEF-H1 is a target of FTO, we conducted a MeRIP assay. Consistent with the MeRIP-seq data, overexpression of FTO decreased the m6A modification level of *GEF-H1* mRNA (Fig. 5H). Moreover, MeRIP-seq data analysis revealed three potential m6A sites within the 3'UTR of *GEF-H1* mRNA. On the basis of these predicted m6A modification sites, we constructed four pmirGLO vectors, including the wild-type and three mutants with site-directed mutations at positions GGACU, GAACU, and GGACA, to assess how m6A modifications affect *GEF-H1* expression via a dual-luciferase reporter assay. For the mutant form of *GEF-H1*, we replaced the adenosine base in the m6A consensus sequence with thymine to abolish the m6A modification (Fig. 5I). The results revealed that mutations 1 and 2 increased luciferase activity compared with that of the wild-type and mutation 3, indicating that *GEF-H1* is under the control of FTO-associated m6A modification at site 3 (Fig. 5J).

We further investigated the mechanism by which m6A modification regulates GEF-H1 expression. Since m6A methylation appears to inhibit GEF-H1 expression, we hypothesized that GEF-H1 transcripts are targets of YTHDF1, an m6A reader protein that promotes the decay of m6A-methylated transcripts (Zaccara and Jaffrey 2020). In addition, short interfering RNA (siRNA)-mediated knockdown of *YTHDF1* in HESCs increased the mRNA and protein levels of GEF-H1 (Fig. 5K, L). An RNA immunoprecipitation (RIP) assay confirmed the interaction between YTHDF1 and *GEF-H1*, providing evidence of their interaction (Fig. 5M). We subsequently performed an RNA degradation assay to investigate whether YTHDF1 promotes the degradation of the *GEF-H1* transcript, and indeed, the *GEF-H1* transcript showed decreased RNA decay rates upon the knockdown of YTHDF1 (Fig. 5N). Thus, our findings revealed that FTO-mediated m6A modification promotes GEF-H1 expression via a m6A-YTHDF1-dependent pathway.

### GEF-H1 mediates the effects of m6A methylation on HESCs migration and invasion

**Fig. 6:**
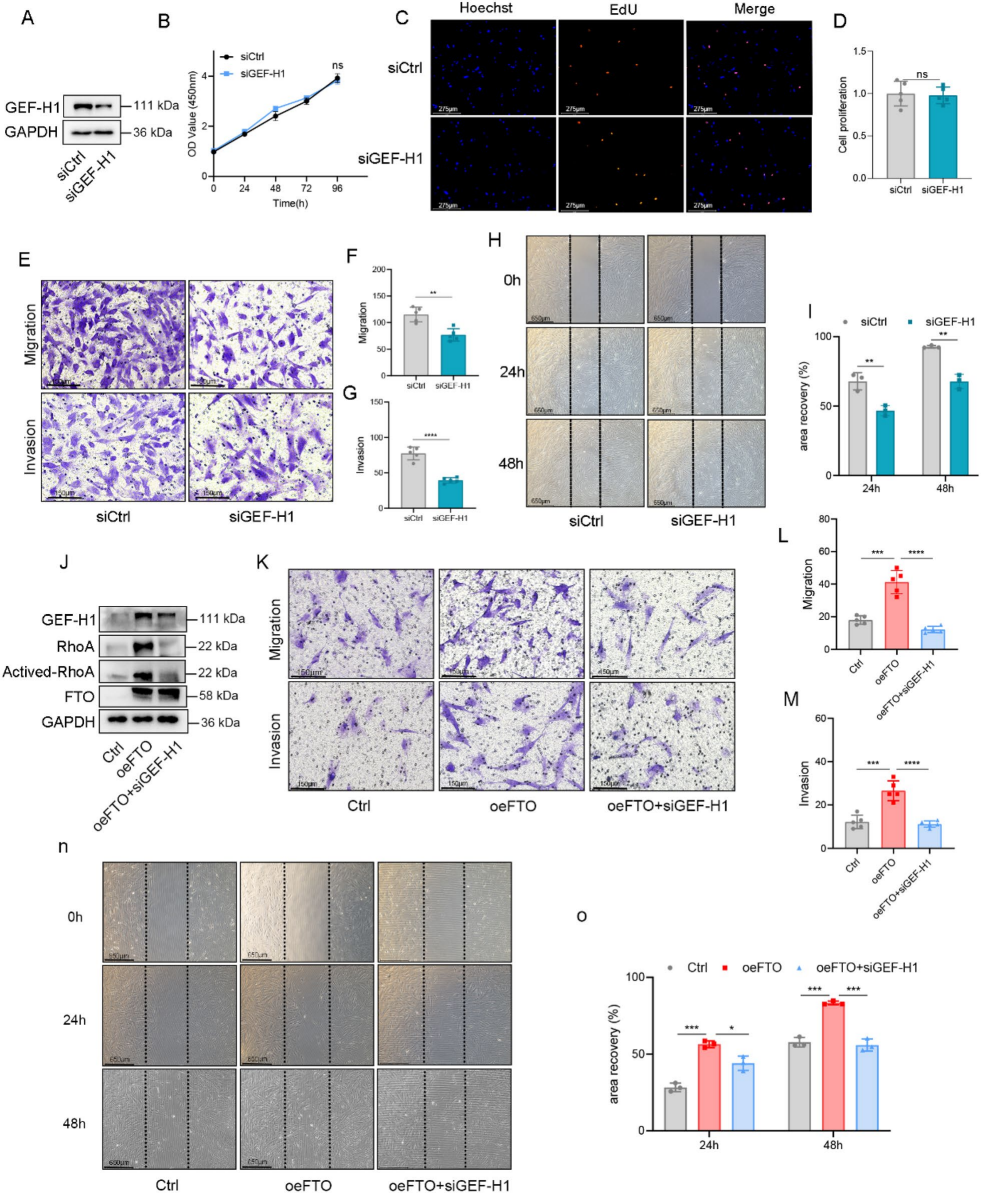
GEF-H1 mediates the effects of m6A methylation on HESCs migration and invasion. **A** Effect of GEF-H1 knockdown in HESCs. **B** Cell proliferation of siCtrl (negative control) and siGEF-H1 (silencing of *GEF-H1*) HESCs was measured via a CCK-8 assay. **C**, **D** EdU assay in HESCs with siCtrl or siGEF-H1 (GEF-H1-knockdown) and quantification of the EdU results. Scale bars: 275 μm. **E**–**G** Transwell migration and invasion assays of HESCs with siCtrl and siGEF-H1. Scale bars: 275 μm. **F**, **G** Cells exhibiting migration/invasion are presented in the histogram. **P < 0.005, ****P < 0.0001. **H**, **I** A wound healing assay was performed on HESCs with knockdown of GEF-H1. A scratch was created, and the width was measured 24 h and 48 h after the scratch. ***P < 0.001, ****P < 0.0001. Scale bars: 650 μm. **J** The efficacy of oeFTO + siGEF-H1 (which inhibits GEF-H1 in HESCs with stable overexpression of FTO) and downstream genes was examined by immunoblotting. **K**‒**M** The invasive and migratory abilities of ctrl, oeFTO and oeFTO + siGEF-H1 HESCs were explored via transwell assays (**K**) and quantitative analysis (**L**-**M**). Scale bars: 275 μm. ***P < 0.001, **** P < 0.0001. (N–O) Representative images and quantification of the migration of GEF-H1-knockdown HESCs or their corresponding controls. Scale bars: 650 μm. *P < 0.05, ***P < 0.001

To elucidate the functions of GEF-H1, we used siRNA to silence GEF-H1 expression and then assessed the pathological phenotypes of HESCs (Fig. 6A). Our findings revealed that siRNA-mediated silencing of GEF-H1 inhibited cell migration and invasion, albeit without affecting cell proliferation (Fig. 6B–I). To determine whether GEF-H1 mediated m6A-dependent migration and invasion in HESCs, we suppressed GEF-H1 expression in HESCs stably overexpressing FTO and observed concurrent inhibition of the activity and expression of RhoA (Fig. 6J). As depicted in Fig. 6K–O, the suppression of GEF-H1 mitigated the increased levels of cell migration and invasion induced by FTO overexpression. Collectively, these findings underscore that FTO-mediated m6A promotes HESCs migration and invasion through its effects on GEF-H1.

## Discussion

**Fig. 7:**
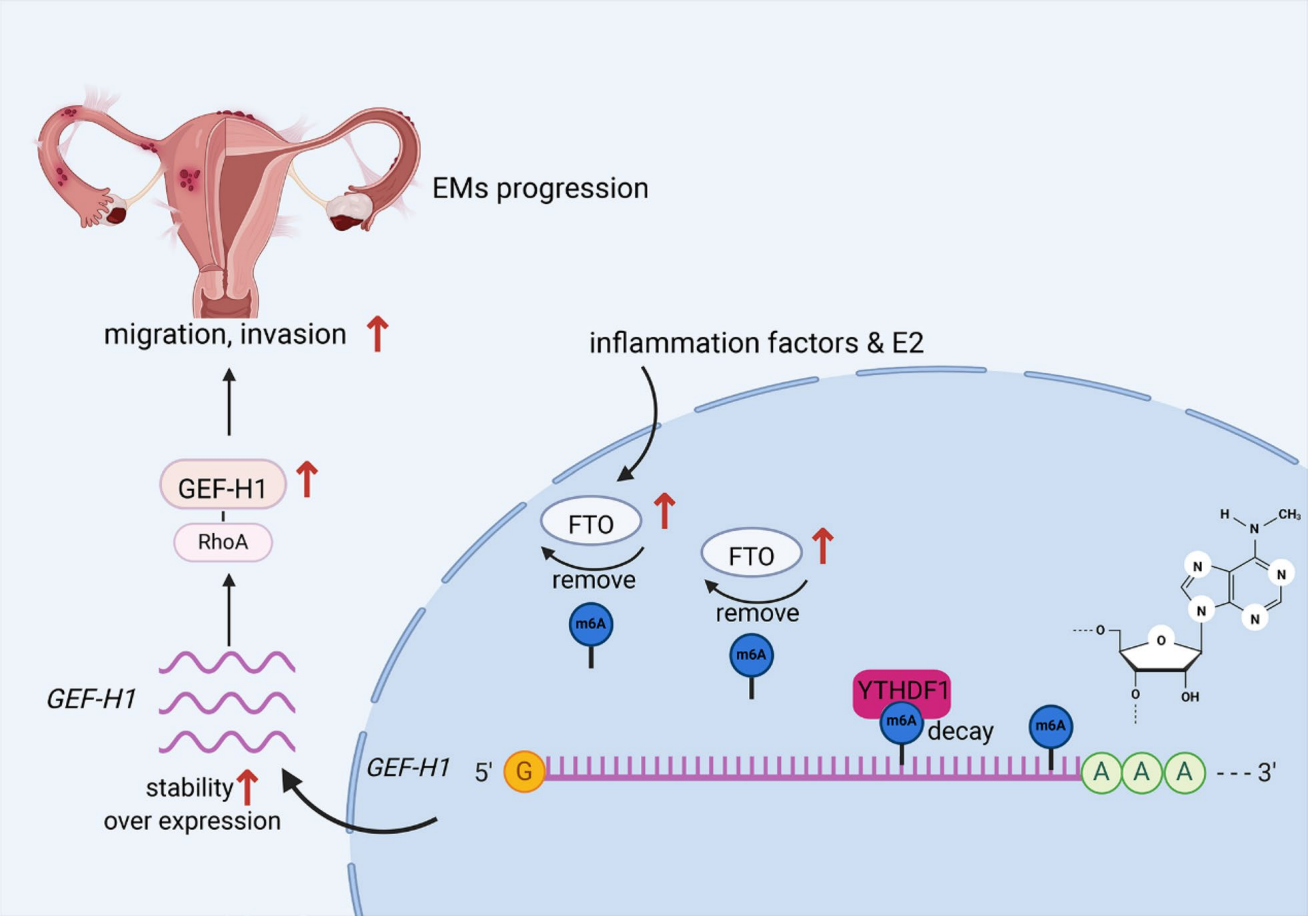
Molecular mechanism by which FTO regulates GEF-H1 degradation through m6A modification, promoting migration and invasion in endometriosis

In this study, we identified a novel role for the obesity-associated protein FTO, which is known primarily for its function as an m6A eraser, in the pathogenesis of endometriosis (Fig. 7). Our findings revealed elevated levels of FTO in endometriosis, leading to a reduction in total m6A levels, thus indicating its substantial involvement in disease progression. Importantly, the modulation of m6A levels by FTO contributes to increased proliferation, migration, and invasion of endometrial stromal cells as well as eutopic and ectopic endometrial primary cells in vitro, ultimately promoting ectopic lesion growth in vivo. Furthermore, we demonstrated that the expression of FTO and its effect on m6A levels are regulated by estradiol (E2) and inflammatory factors, which are key drivers of endometriosis pathogenesis. These findings suggest that FTO-mediated alterations in m6A levels represent a crucial downstream mechanism contributing to the pathogenic behavior of endometrial stromal cells.

At the molecular level, *FTO* overexpression leads to a reduction in m6A enrichment in the 3′UTRs of mRNAs across the transcriptome. At the pathway level, *FTO* overexpression alters m6A enrichment in key genes involved in the GTPase pathway, including *GEF-H1*, *RhoA*, *ROCK*, *RAC1*, *PI3K*, and *Cdc42*. Furthermore, we observed that in *FTO*-overexpressing cells, GEF-H1 expression is regulated by the m6A reader YTHDF1. Consistently, the knockdown of YTHDF1 led to an increase in the protein level of GEF-H1. The increase in GEF-H1 expression induced by FTO-mediated m6A modification also promoted the expression of downstream factors, thereby activating the RhoA pathway. Finally, we found that GEF-H1 plays an important role in mediating the migration and invasive functions of HESCs, contributing to the progression of endometriosis. Collectively, our findings underscore the critical role of FTO-mediated m6A regulation in the pathogenesis of endometriosis.

m6A is the most common internal modification of mRNA molecules and influences various physiological and pathological pathways by modulating the mRNA fate. FTO, the pioneering demethylase acknowledged for its recognition of m6A, has been implicated in human diseases, notably breast cancer, by fostering cell proliferation and metastasis, as evidenced in both in vitro and in vivo studies (Niu et al. 2019). E2-induced FTO, which is mediated through the activation of the PI3K/AKT and MAPK signaling pathways, has been shown to increase the proliferation and invasion of endometrial cancer cells (Zhang et al. 2012). In specific subtypes of acute myeloid leukemia (AML), elevated FTO levels decrease m6A levels in ASB2 and RARA mRNA transcripts, thereby promoting their expression. This, in turn, hampers all-trans retinoic acid (ATRA)-induced differentiation of AML cells (Li et al. 2017). FTO also plays critical roles in cancer stem cell self-renewal and immune evasion (Su et al. 2020). To the best of our knowledge, only two studies involving the relationship between m6A modifications and endometriosis have been reported. A study by Jiang et al. analyzed 20 m6A regulators and reported that most of these regulators were significantly dysregulated in endometriosis, with some proposed as diagnostic biomarkers (Jiang et al. 2020). Alternatively, Li et al. reported that METTL3 was downregulated in endometriosis, resulting in increased cellular migration and invasion through *microRNA126*, which is regulated by DGCR8 in m6A-dependent manner (Li et al. 2021). Notably, consistent with our study, Li et al. also reported decreased levels of m6A in endometriosis, but their observation of METTL3 downregulation contrasts with our findings. The reason for this discrepancy is unclear but could be attributed to the different inclusion criteria of the clinical samples used. Wang et al. discovered that the decreased expression of FTO in EMs influences the glycolysis, proliferation, and metastasis of ectopic endometriotic stromal cells (Ec-ESCs) by targeting ATG5, a finding that diverges from our own findings (Wang et al. 2022). This discrepancy may arise from variations in primary cell lines derived from the human endometrial stroma. To reconcile this, we utilized three distinct cell lines and employed two different in vivo experimental methodologies to substantiate the impact of FTO on EMs, thereby increasing the robustness of our findings. Despite these observations, there remains limited mechanistic evidence elucidating how dysregulated m6A methylation specifically contributes to endometriosis. Presenting novel insights, we show that elevated levels of FTO lead to reductions in m6A mRNA methylation, thereby fostering the pathogenic characteristics of endometrial cells via activation of the RhoA pathway. However, while the influence of FTO on migration and invasion appears to be mediated through the RhoA upstream regulator GEF-H1, other effects on proliferation and apoptosis were unrelated to GEF-H1. Consequently, the exploration of these alternative mechanisms warrants further investigation.

Endometriosis is strongly correlated with elevated levels of E2 and chronic inflammation. Women afflicted with endometriosis often exhibit heightened circulating and local concentrations of E2, alongside concurrent chronic pelvic inflammation characterized by elevated plasma interleukin-1β levels (Marquardt et al. 2019; Peng et al. 2020). One study reported that increases in the m6A levels in *ERRγ* mRNA (estrogen receptor-related receptor) could trigger the splicing of precursor *ESRRG* mRNA to promote its expression, with the upregulation of ERRγ conferring chemoresistance to cancer cells through the upregulation of ABCB1 and CPT1B (Shen et al. 2021). Another study indicated that METTL14 promotes FOXO1 expression through increased m6A modification, thereby facilitating TNF-α-induced inflammation in endothelial cells (Jian et al. 2020). Numerous other studies have explored the interplay among E2, inflammatory factors, and m6A modifications (Jian et al. 2020; Yang et al. 2024). In our study, we found that both E2 and inflammatory factors can regulate the expression of m6A regulatory factors, leading to decreased m6A levels in HESCs. These findings suggest a novel mechanism by which E2 and inflammation mediate endometriosis, which warrants further exploration in subsequent studies.

Rho family GTPases regulate cell migration and invasion through cooperative reorganization of actin and the microtubule cytoskeleton, as well as the turnover of cell-substrate adhesions (Ridley 2001; Etienne-Manneville and Hall 2002). Rho GTPases also govern cell attachment, spreading, and detachment processes (Ridley et al. 2003). In this study, GO and KEGG analyses revealed significant enrichment of m6A-modified mRNAs associated with Rho family GTPases, along with downstream pathways. These findings suggest a regulatory role of m6A modifications in the modulation of Rho family GTPases. In addition, knockdown of *METTL3* and *METTL14* in HESCs increased AKT phosphorylation at Ser473 and activated the AKT pathway, which is downstream of GEF-H1 (Liu et al. 2018). Furthermore, we found that FTO-mediated m6A mRNA methylation regulates the RhoA pathway, thereby influencing cell migration and invasion in endometriosis. On the basis of these findings, other endometriosis-related mechanisms, such as adhesion and EMT, may also be under the control of m6A mRNA methylation. Indeed, our findings may also be applicable to other diseases driven by increased Rho family signaling.

In summary, we present compelling evidence supporting the upregulation of FTO and its functional relevance in endometriosis. FTO-mediated m6A mRNA methylation facilitates the proliferation, migration, and invasion of HESCs. These effects are underscored by diminished m6A mRNA methylation in *GEF-H1* mRNA, resulting in augmented *GEF-H1* expression via evasion of YTHDF1-mediated mRNA decay. Consequently, increased GEF-H1 levels stimulate the migration and invasion of HESCs via the modulation of the RhoA pathway. Our findings not only shed new light on the pathogenesis of endometriosis but also open avenues for the development of optimized therapeutic strategies.

## Conclusions

This study establishes FTO as a critical factor in EMs, revealing its role in increasing GEF-H1 expression through m6A methylation modulation. These findings highlight how FTO-driven pathways contribute to the aggressive behavior of endometrial stromal cells, emphasizing the need for targeted therapies. By focusing on FTO, we can potentially develop innovative strategies to improve treatment outcomes for patients with EMs.

## Supplementary Information

Supplementary Material 1. Figure S1. The Construction and Identification of an EMs Mouse Model and Bioinformatic Analysis of EMs-Related Data. (A) Establishment of an endometriotic mouse model. (B) Tissue sections of ectopic lesions in EMs model mice. (C) Analysis of modification-related gene expression in Normal (n = 34), Eutopic (n = 127) and Ectopic (n = 45) according to the GEO database. *P < 0.05, **P < 0.005, ***P < 0.001, ****P < 0.0001

Supplementary Material 2. Figure S2. The impact of FTO on the proliferation, migration, and invasion of Eu-ESCs and Ec-ESCs. (A-B) Cell proliferation of Eu-ESCs overexpressing FTO (A) and suppressing FTO (B) was measured by a CCK-8 assay. **P < 0.005, ****P < 0.0001. (C-E) The effects of FTO overexpression on the migration and invasion of Eu-ESCs were validated through transwell assays. **P < 0.005, Scale bars: 150 μm. (F–H) Validation of the impact of FTO downregulation on the migration and invasion of Eu-ESCs using transwell assays. **P < 0.005, ***P < 0.001, Scale bars: 150 μm. (I-J) The effects of FTO overexpression (I) and suppression (J) on the proliferation of Ec-ESCs were assessed using CCK-8 assays. ****P < 0.0001. (K‒M) Transwell assays confirming the promotion of migration and invasion in Ec-ESCs by FTO overexpression. **P < 0.005, Scale bars: 150 μm. (N‒P) Transwell assay validating the impact of FTO knockdown on the migration and invasion of Ec-ESCs. **P < 0.005, Scale bars: 150 μm

## Data Availability

The datasets used and analysed during the current study are available from the corresponding author on reasonable request.

## Abbreviations

EMs: Endometriosis
m6A: N6-methyladenosine
HESCs: Human endometrial stromal cells
Eu-ESCs: Primary eutopic endometrial stromal cells
Ec-ESCs: Primary ectopic endometrial stromal cells
FTO: Fat mass and obesity-associated protein
GEF-H1: Rho guanine nucleotide exchange factor
RhoA: Ras homolog family member A
RAC1: Rac family small GTPase 1
CDC42: Cell division cycle 42
AKT: AKT serine/threonine kinase
LIMK: LIM domain kinase 1
YTHDF1/2: YTH N6-methyladenosine RNA binding protein F1/2
E2: Estrogen
METTL3/14: Particular, methyltransferase-like 3/14
ALKBH5: AlkB homolog 5
Normal: Normal endometrial tissues
Eutopic: Eutopic endometrial tissues
Ectopic: Ectopic endometrial tissues
shRNA: Short hairpin RNA
siRNA: Short interfering RNA
LPS: Lipopolysaccharide
IL-1β: Interleukin-1 beta
