# Supplementary figures and images for: A novel mechanism of FTO modulating the progression of endometriosis through mediating the m6A methylation of GEF-H1 in a YTHDF1-dependent manner
Source: Mol Med. 2025 Feb 25;31:78. doi: 10.1186/s10020-025-01130-8 (PMC11863856; doi:10.1186/s10020-025-01130-8)

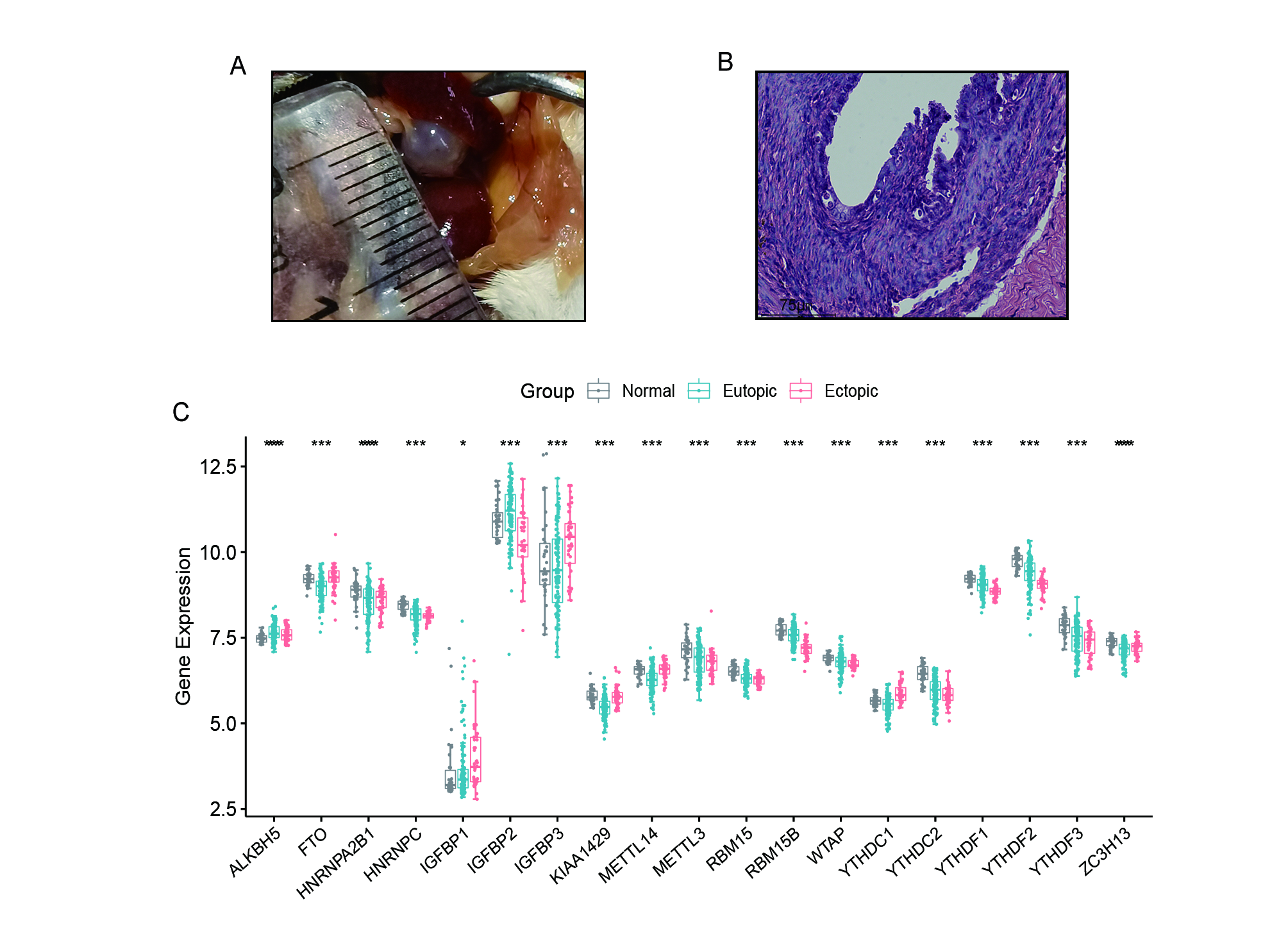

Supplement: Supplementary file 1 — Supplementary Material 1. Figure S1. The Construction and Identification of an EMs Mouse Model and Bioinformatic Analysis of EMs-Related Data. (A) Establishment of an endometriotic mouse model. (B) Tissue sections of ectopic lesions in EMs model mice. (C) Analysis of modification-related gene expression in Normal (n = 34), Eutopic (n = 127) and Ectopic (n = 45) according to the GEO database. *P < 0.05, **P < 0.005, ***P < 0.001, ****P < 0.0001 [file 10020_2025_1130_MOESM1_ESM.tif]

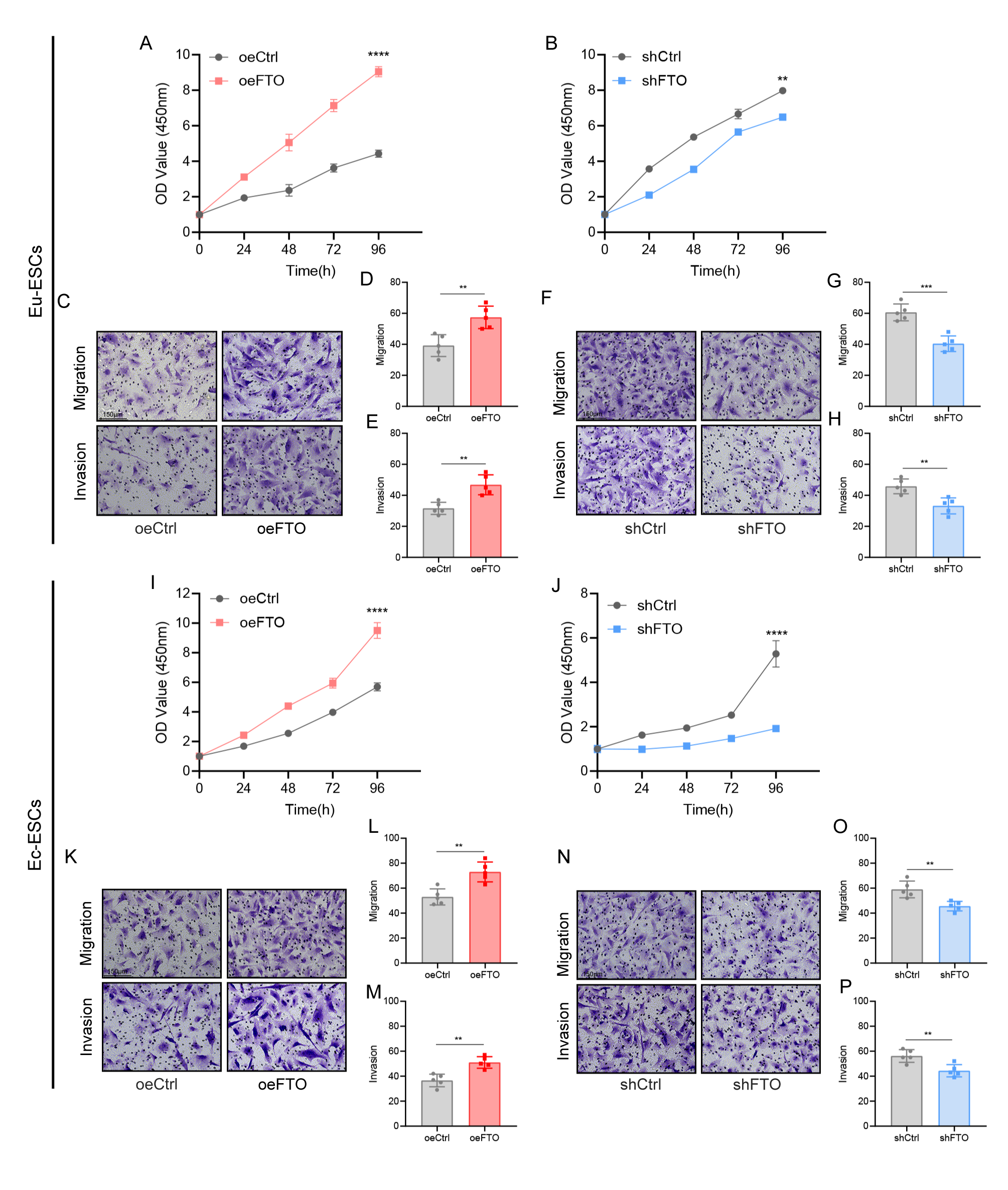

Supplement: Supplementary file 2 — Supplementary Material 2. Figure S2. The impact of FTO on the proliferation, migration, and invasion of Eu-ESCs and Ec-ESCs. (A-B) Cell proliferation of Eu-ESCs overexpressing FTO (A) and suppressing FTO (B) was measured by a CCK-8 assay. **P < 0.005, ****P < 0.0001. (C-E) The effects of FTO overexpression on the migration and invasion of Eu-ESCs were validated through transwell assays. **P < 0.005, Scale bars: 150 μm. (F–H) Validation of the impact of FTO downregulation on the migration and invasion of Eu-ESCs using transwell assays. **P < 0.005, ***P < 0.001, Scale bars: 150 μm. (I-J) The effects of FTO overexpression (I) and suppression (J) on the proliferation of Ec-ESCs were assessed using CCK-8 assays. ****P < 0.0001. (K‒M) Transwell assays confirming the promotion of migration and invasion in Ec-ESCs by FTO overexpression. **P < 0.005, Scale bars: 150 μm. (N‒P) Transwell assay validating the impact of FTO knockdown on the migration and invasion of Ec-ESCs. **P < 0.005, Scale bars: 150 μm [file 10020_2025_1130_MOESM2_ESM.tif]
